# Closing the gap: A workflow-based analysis of postconsent failure to restore hospital autopsy utilization

**DOI:** 10.1016/j.acpath.2026.100300

**Published:** 2026-09-23

**Authors:** Alejandro Partida Contreras, John Darcy, Roberto Gamez, Jude Abadie

**Affiliations:** Department of Pathology, Texas Tech University Health Sciences Center El Paso, El Paso, TX, USA

**Keywords:** Hospital autopsy, Pathology education, Quality improvement, Workflow analysis

## Abstract

Hospital autopsy rates continue to decline despite their established role in diagnostic quality assurance, patient safety, and medical education. At our academic institution, autopsy utilization decreased to less than 1% of inpatient deaths. We hypothesized that this decline reflects failures within the autopsy workflow rather than limitations in consent acquisition. We analyzed 4229 inpatient deaths from 2019 to 2025 and performed a detailed audit of 140 autopsy consents to evaluate documentation quality, accuracy of indication, and involvement of pathology personnel. In addition, clinical resident physicians were surveyed using a six-scenario assessment to evaluate recognition of autopsy indications. Autopsy utilization declined from 3.9% to 0.57%, while the consent-to-autopsy conversion rate decreased from 52% to 8%, indicating substantial loss of cases after consent had been obtained. Forty percent of consents were incomplete or inaccurate, and pathology personnel were involved in only 1.4% of cases. Survey results demonstrated variable recognition of autopsy indications and infrequent consideration of autopsy in clinical practice. A workflow-based model showed that targeted improvements in consent quality and initiation could increase autopsy utilization up to 4.4-fold. These findings indicate that autopsy underutilization reflects modifiable system-level inefficiencies rather than solely cultural or financial barriers. A structured, workflow-based approach provides a reproducible framework to improve autopsy utilization and reestablish its role in quality assurance and medical education.

## Introduction

The hospital autopsy has long been regarded as one of the most powerful tools for understanding disease, evaluating diagnostic accuracy, and advancing medical education. In the first half of the twentieth century, autopsy rates in major academic hospitals exceeded 50%, and in some institutions approached 90%, reflecting its central role in clinical practice and its function as a benchmark of institutional quality.^1^ The autopsy provides a unique opportunity for direct clinicopathologic correlation, allowing clinicians to assess the accuracy of premortem diagnoses, identify unexpected findings, and refine future patient care. During the past several decades, however, hospital autopsy rates have declined dramatically in the United States and worldwide, with contemporary utilization now estimated at <5% of inpatient deaths at most institutions.^1^, ^2^, ^3^, ^4^

This decline has occurred despite consistent evidence that the autopsy continues to reveal clinically significant discrepancies between premortem and postmortem diagnoses. Multiple studies conducted across different eras of medical practice have demonstrated major diagnostic discrepancies in approximately 20–30% of cases, including conditions that, if recognized earlier, might have altered clinical management or survival.^2^^,^^3^ These findings have remained relatively stable despite advanced imaging and laboratory testing, reinforcing the persistent diagnostic and educational value of autopsy in modern medical practice.^5^^,^^6^ This emphasizes the continued relevance of autopsy as a tool for quality assurance and patient safety. In addition to its diagnostic value, the autopsy remains essential for medical education, providing trainees with a comprehensive understanding of disease processes, procedural complications, and the limitations of current diagnostic technologies.^4^

Numerous explanations have been proposed for the decline in hospital autopsy utilization. Structural factors include the elimination of minimum autopsy rate requirements by accrediting organizations, lack of direct reimbursement, increasing clinical workload, and the perception that modern diagnostic technologies have rendered autopsy obsolete.^1^^,^^4^ Cultural factors, including clinician discomfort with requesting consent and concerns about causing distress to families, have also been cited as barriers. Surveys of physicians-in-training at academic institutions have consistently demonstrated substantial knowledge gaps regarding autopsy indications, the consent process, and the clinical value of postmortem examination, with few programs providing formal instruction on how to request an autopsy or how to discuss autopsy topics with the next of kin.^7^ These findings indicate that even in teaching hospitals, where autopsy historically played a central role, the process is often poorly understood.

In academic teaching centers, hospital autopsies remain uncommon despite their well-established educational and quality-assurance value. Surveys of teaching hospitals have demonstrated substantial deficiencies in autopsy consent practices, limited physician education regarding autopsy, and the need for standardized institutional consent processes to improve autopsy utilization.^8^ These observations indicate that institutional organization, standardized consent practices, and clinician engagement are critical determinants of autopsy utilization. Despite the practical constraints of modern health care, substantially higher autopsy rates can be achieved when autopsy is systematically offered, clinicians are trained to recognize appropriate indications, and the consent process is supported by pathology services.

Rather than interpreting the decline in autopsy rates as a single outcome, the present study approaches autopsy utilization as a multistep clinical workflow. Completion of a hospital autopsy requires sequential processes, including recognition of an appropriate indication, initiation of the consent discussion, accurate documentation, and successful performance of the examination. Failure at any stage results in loss of the autopsy opportunity. This process-oriented perspective allows identification of specific, potentially modifiable points of failure within the system.

The present study was designed as a comprehensive quality-improvement initiative to evaluate the hospital autopsy pathway in a tertiary academic teaching facility. The objectives were: first, to quantify temporal trends in autopsy utilization over a seven-year period; second, to identify system-level failures in autopsy initiation, consent quality, and conversion to completed autopsy using a case-level consent database; and third, to construct a predictive model estimating the expected effect of a targeted educational intervention on future autopsy performance. This study provides a reproducible model for restoring hospital autopsy utilization in academic medical centers.

## Materials and methods

This study was conducted as a single-center quality improvement initiative at a tertiary academic teaching hospital in El Paso, Texas (University Medical Center of El Paso), to evaluate the hospital autopsy workflow and identify modifiable institutional factors contributing to autopsy underutilization. The project was designed as a multistage investigation consisting of a retrospective baseline analysis of autopsy utilization and consent practices, a cross-sectional assessment of clinical resident physician's knowledge and perceptions regarding hospital autopsy, the development of a predictive workflow-based model of autopsy performance, and the implementation of a targeted educational intervention with planned prospective validation. The study was reviewed by the Texas Tech University Health Sciences Center El Paso Institutional Review Board (IRB) and was determined to meet criteria for quality improvement with exemption from full review (IRB protocol number E26022).

At our institution, autopsy consent discussions and documentation were performed primarily by clinical residents and, less frequently, by advanced practice providers. Attending physicians were rarely directly involved in obtaining consent during the study period. No formalized training program existed for clinicians regarding autopsy indications or the consent process, and responsibility for initiating and completing consent documentation was largely dependent on individual clinical teams.

The retrospective baseline period included all inpatient deaths occurring between January 1, 2019 and December 31, 2025. Institutional mortality records were used to obtain the total number of inpatient deaths per year, the number of hospital autopsies performed, and the number of autopsy consents obtained. These data were used to calculate the annual hospital autopsy rate, defined as the number of autopsies performed divided by the total number of inpatient deaths, the consent initiation rate, defined as the number of autopsy consents obtained divided by the total number of inpatient deaths, and the consent-to-autopsy conversion rate, defined as the number of autopsies performed divided by the number of consents obtained.

Pediatric and neonatal patients were excluded based on predefined inclusion criteria, which were limited to adult inpatient deaths. Additionally, pediatric patients in our region are primarily managed at El Paso Children's Hospital, which operates as a separate institution from University Medical Center of El Paso. As a result, pediatric cases were not included in the analysis, and pediatric residents were not surveyed, as they are not involved in the hospital autopsy consent process within the study setting.

A detailed analysis of the autopsy consent process was performed using a manually curated database of all available hospital autopsy consents from January 1, 2023 through December 31, 2025. For each case, demographic and clinical variables were recorded, including year and month of death, patient age and sex, the clinical service responsible for obtaining consent, and the role of the clinician completing the consent form. Process-related variables included whether the consent form was completed in its entirety, the accuracy and completeness of the documentation, whether institutional criteria for hospital autopsy were met, whether the case fell under the jurisdiction of the medical examiner, whether pathology personnel were involved in the consent process, and the final disposition of the autopsy request. Institutional indications for hospital autopsy were determined using established clinical criteria. To evaluate the reliability of the consent process, we developed an integrated measure of consent quality. Consents were classified as high quality when the form was completed in full, the determination of autopsy indication correctly met institutional criteria, and the clinical reasoning was appropriately documented. Consents were classified as low quality when documentation was incomplete and/or the indication for autopsy was incorrectly determined.

Clinical resident physician knowledge and perceptions regarding hospital autopsy practice were evaluated using an anonymous, voluntary questionnaire (Supplemental Material 1) distributed to residents in internal medicine, neurology, and surgery. The survey collected demographic data, including specialty and postgraduate year of the residents. Furthermore, it assessed attitudes toward the educational and clinical value of autopsy, the frequency with which clinical residents considered requesting an autopsy in routine practice, and perceived institutional and national factors contributing to declining autopsy rates. The questionnaire also evaluated prior exposure to autopsy during training and understanding of the legal requirement for consent for hospital autopsy outside of medical examiner jurisdiction.

Objective knowledge was assessed using two components: recognition of institutional indications for hospital autopsy and responses to six scenario-based clinical vignettes representing common clinical situations in which autopsy may or may not be indicated. For each participant, a knowledge score was calculated as the number of correctly classified scenarios (range: 0–6). Survey responses were collected without identifiers, and participation was voluntary.

The hospital autopsy process was conceptualized as a multistep workflow consisting of inpatient death, initiation of the consent process, completion of a valid consent, and performance of the autopsy (Fig. 1). The overall hospital autopsy rate was modeled as the product of two sequential workflow parameters: the consent initiation rate (number of autopsy consents divided by the number of inpatient deaths) and the consent-to-autopsy conversion rate (number of autopsies performed divided by the number of consents obtained). This approach allowed quantitative identification of the stages at which cases were lost within the autopsy pathway.

**Fig. 1:**
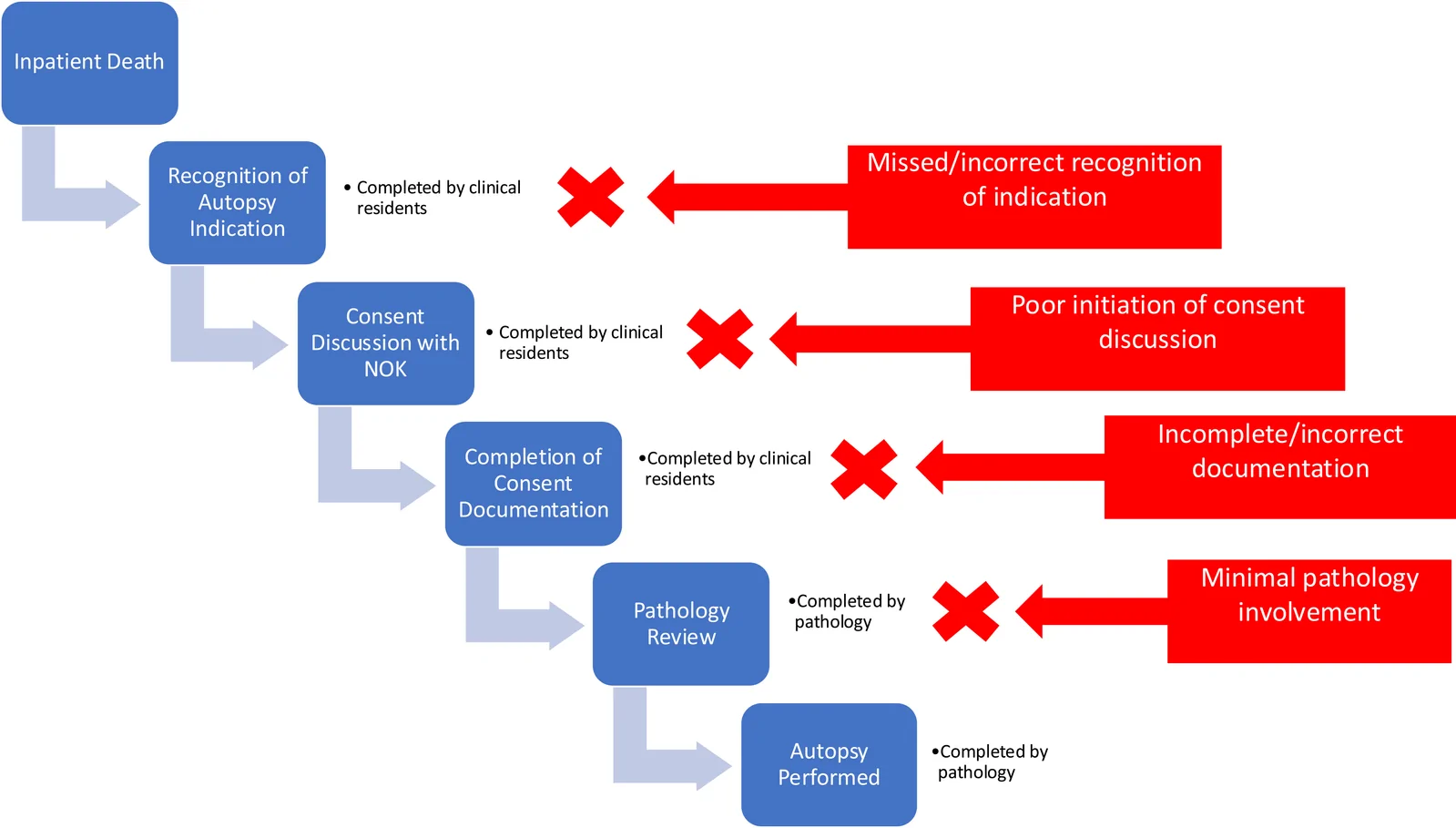
Autopsy workflow and points of case attrition. Schematic representation of the hospital autopsy process as a sequential workflow, including inpatient death, recognition of autopsy indication, consent discussion with next of kin, completion of consent documentation, pathology review, and performance of the autopsy. Key points of case attrition identified in this study are highlighted, including missed or incorrect recognition of indication, poor initiation of consent discussions, incomplete or incorrect documentation, and minimal pathology involvement. NOK, next of kin.

A scenario-based predictive model of hospital autopsy utilization was constructed using institutional baseline data. The autopsy rate was expressed as the product of the consent initiation rate and the consent-to-autopsy conversion rate:

Autopsyrate=(consents÷inpatientdeaths)×(autopsies÷consents).

This framework conceptualized the autopsy process as a sequential workflow consisting of inpatient death, initiation of the consent process, completion of valid documentation, and performance of the autopsy. Scenario-based projections were generated by modifying the consent initiation and consent-to-autopsy conversion parameters to estimate the potential impact of targeted improvements in clinician recognition of autopsy indications and documentation accuracy. A benchmark autopsy rate of 5%, corresponding to contemporary academic centers, was used as a reference target.

The primary outcome measure was the hospital autopsy rate. Secondary outcome measures included the consent initiation rate, the consent-to-autopsy conversion rate, the proportion of high-quality consents, the frequency of pathology involvement in the consent process, and resident physician knowledge and perception scores. Data analysis was primarily descriptive and focused on characterizing workflow performance in routine clinical practice. Results are presented as counts, proportions, and summary measures to illustrate patterns in autopsy utilization, consent quality, and resident knowledge. The composite consent quality metric provided an interpretable indicator of documentation performance at the case level, and resident knowledge was expressed as a total scenario-based score.

## Results

A total of 4229 inpatient deaths occurred at the study institution between 2019 and 2025. During this period, hospital autopsy utilization declined from 3.9% in 2019 to 0.57% in 2025 despite increasing inpatient mortality. This decline was primarily driven by a marked reduction in the consent-to-autopsy conversion rate, which decreased from 52% to 8% over the same period. From 2021 through 2025, the autopsy rate remained at or near 1%, indicating a sustained and progressive reduction in autopsy utilization (Fig. 2).

**Fig. 2:**
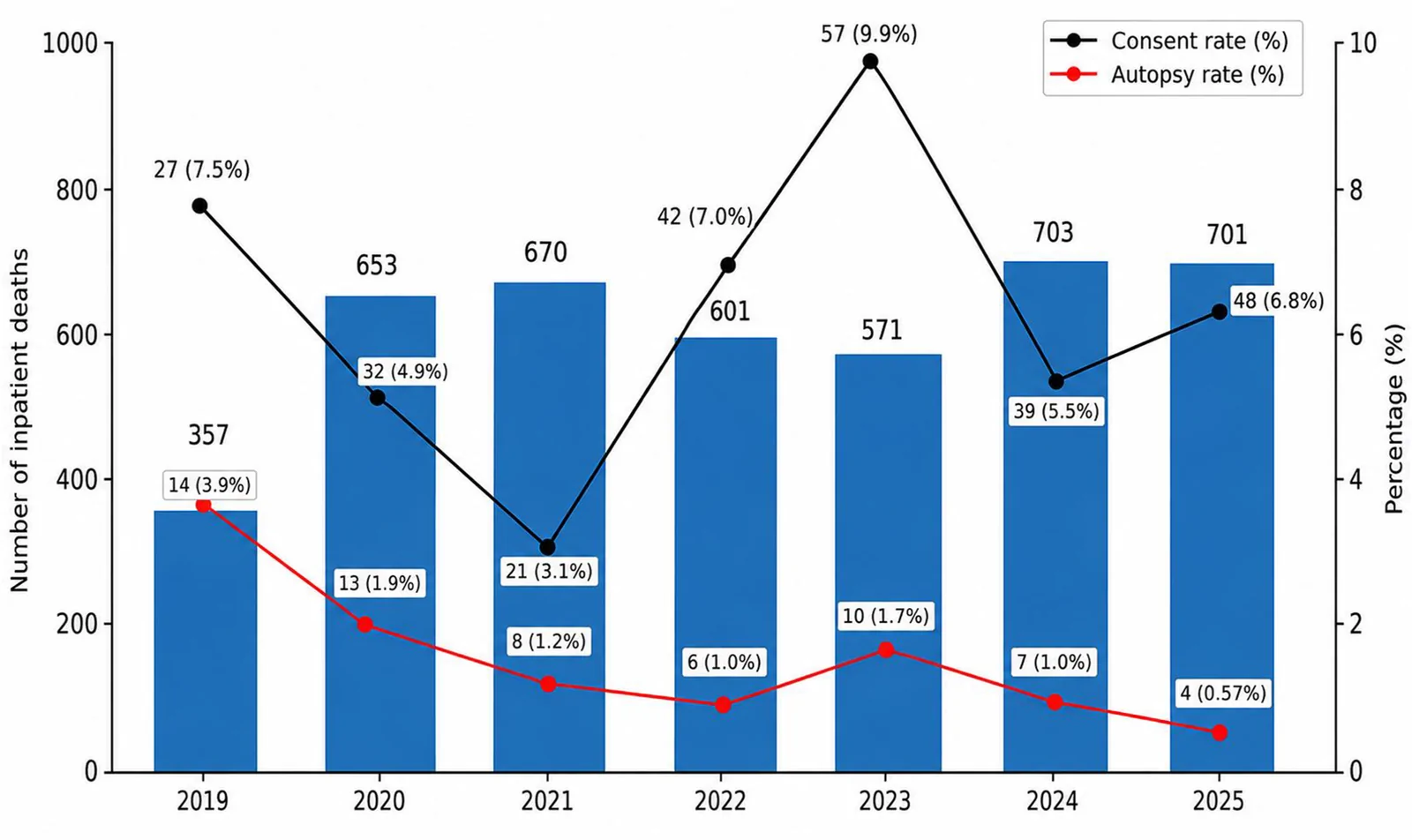
Annual inpatient deaths, autopsy consent rates, and completed hospital autopsy rates at the study institution from 2019 to 2025. Bars represent the total number of inpatient deaths per year. The black line indicates the autopsy consent rate, and the red line indicates the completed hospital autopsy rate. Numerical labels show the number of cases and corresponding percentage relative to total inpatient deaths for each year.

To determine where autopsy opportunities were missed, we examined the autopsy pathway as a stepwise clinical process, beginning with an inpatient death, presence or absence of a consent discussion, and ending with the completion of the autopsy after signed consent. The consent initiation rate remained consistently low throughout the study period, ranging from 6.8% to 9.1% of inpatient deaths. This year-to-year variation did not parallel the marked decline observed in the overall autopsy rate. In contrast, the consent-to-autopsy conversion rate progressively declined over the study period, falling from 52% in 2019 (14 of 27 consents; ratio 14:27) to 41% in 2020 (13 of 32; ratio 13:32)**,** 28% in 2021 (8 of 29; ratio 8:29)**,** 14% in 2022 (6 of 42; ratio 6:42)**,** 12% in 2023 (7 of 57; ratio 7:57)**,** 20% in 2024 (7 of 35; ratio 7:35), and 8% in 2025 (4 of 48; ratio 4:48)**.** This pattern indicates that the greatest loss occurred after consent had been obtained, suggesting missed opportunities during the consent and documentation process. Detailed review of 140 hospital autopsy consents obtained between 2023 and 2025 revealed discrepancies in the reliability of the consent process. Completion of all required elements of the consent form (Supplemental Material 2) was observed in 93 cases (66.4%), whereas 47 consents (33.6%) were incomplete. Common deficiencies included absence of required decedent identifiers (e.g., full name, date of birth, and medical record number), missing clinician signatures, incomplete initial sections of the consent form, and internally inconsistent documentation in determining institutional criteria for autopsy.

Correct and complete documentation of the clinical indication and consent elements was present in 86 cases (61.4%). Using the predefined measure of consent quality, 84 consents (60.0%) were classified as high quality and 56 (40.0%) as low quality. Thus, in approximately two out of every five consent attempts, the autopsy request process failed due to incomplete documentation and/or determination of autopsy indicators. Pathology personnel were rarely involved in the consent process; only 2 of 140 cases (1.4%) involved pathology personnel, indicating that the consent workflow was almost entirely managed without direct pathology participation (Table 1).

**Table 1:** Quality and completeness of hospital autopsy consent documentation (n = 140). Assessment of consent form completion, documentation accuracy, overall quality, and pathology involvement. A substantial proportion of consents were incomplete or incorrectly filled, and pathology participation in the consent process was minimal.

Variable	Result
Total consents reviewed	140
Consent completed	93 (66.4%)
Consent incomplete	47 (33.6%)
Correctly filled	86 (61.4%)
Incorrectly filled	54 (38.6%)
Overall high quality	84 (60.0%)
Overall low quality	56 (40.0%)
Pathology involved	2 (1.4%)

There were striking discrepancies between clinical residents' understanding of autopsy value and decisions to request an autopsy. Among the 40 clinical resident physicians surveyed, the vast majority agreed that hospital autopsy contributes significantly to medical education and the improvement of patient care. Despite this recognition, clinical residents reported that they rarely or only occasionally considered requesting an autopsy in routine clinical practice. When asked to identify the primary factor responsible for the national decline in hospital autopsy rates, the frequently selected responses included financial and/or cultural barriers, lack of consent referral, increased reliance on advanced diagnostic imaging, and other premortem diagnostic modalities (Table 2, Fig. 3).

**Table 2:** Resident physician demographics, knowledge, and perceptions regarding hospital autopsy (n = 40). Distribution of respondents by postgraduate year and specialty with perceived causes of low autopsy utilization and knowledge score. Findings demonstrate variable recognition of autopsy indications despite high perceived educational value.

Variable	Result
*Clinical resident physician demographics*	
PGY-1	18
PGY-2	11
PGY-3	8
PGY-4	3
Specialty – Surgery	18
Specialty – Internal medicine	18
Specialty – Neurology	4
*Knowledge and perceptions*	
Perceived cause – financial & cultural barriers	24 (60.0%)
Perceived cause – cultural barriers & lack of referrals	12 (30.0%)
Perceived cause – high rates of accurately dx	4 (10.0%)
Knowledge score (mean ± SD)	4.15 ± 1.67

**Fig. 3:**
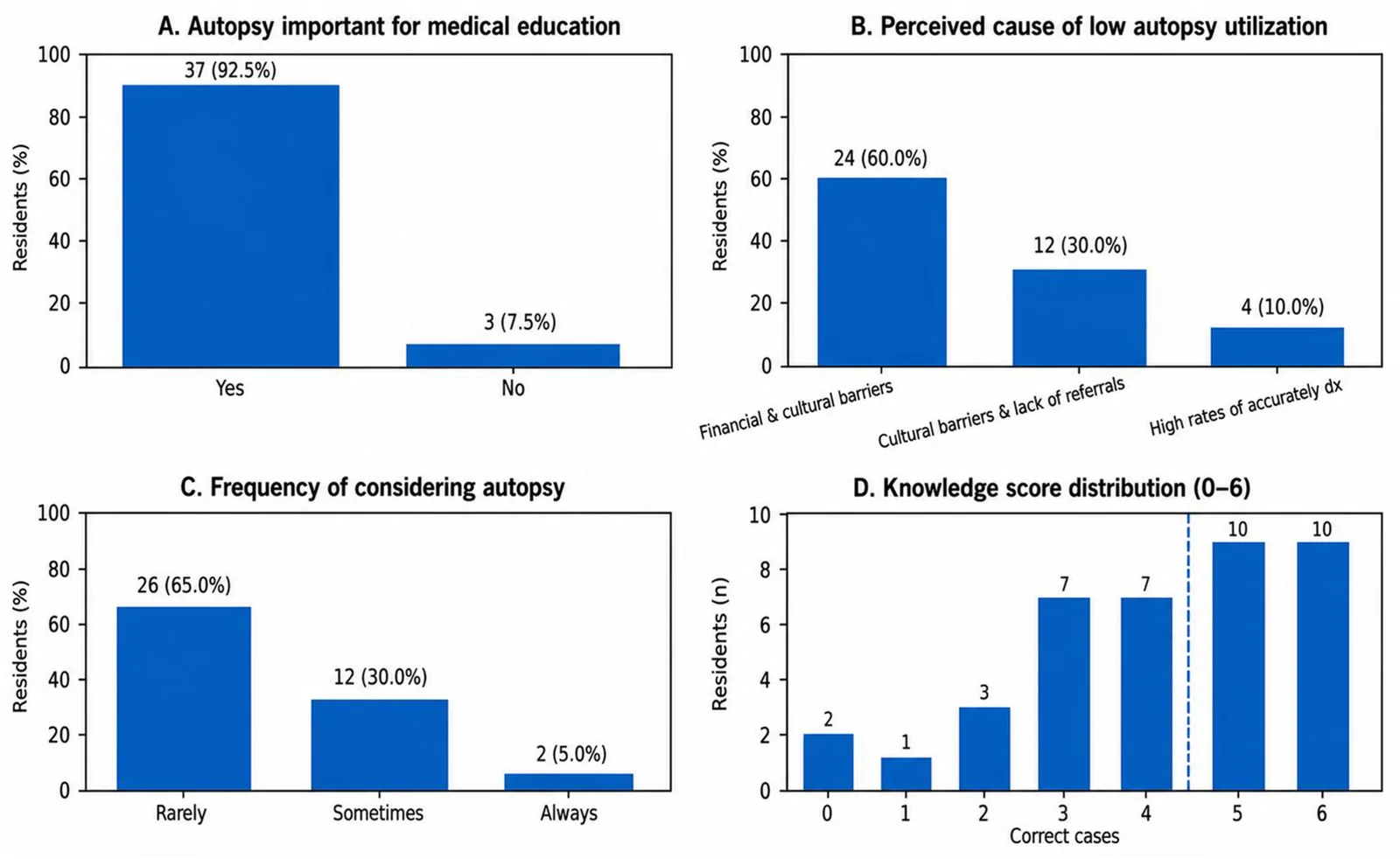
Clinical resident physician questionnaire: perceptions, clinical practice, and knowledge of hospital autopsy (n = 40). **(A)** Proportion of clinical residents who agreed that autopsy contributes to medical education. **(B)** Perceived primary causes of low autopsy utilization. **(C)** Frequency of considering autopsy in routine clinical care. **(D)** Distribution of correct responses to six scenario-based indication questions; the dashed line indicates the mean score. Values are shown as n (%). Despite near-universal recognition of educational value, knowledge and clinical utilization were variable.

Evaluation of hospital clinical resident physicians’ knowledge of criteria for performing an autopsy revealed substantial variability in recognizing appropriate autopsy indications. Performance on the six scenario-based clinical vignettes showed a wide distribution of correct responses, indicating inconsistent application of institutional criteria in common clinical situations. In addition, only a subset of residents correctly identified next of kin authorization as the required legal mechanism for hospital autopsy outside of medical examiner jurisdiction, demonstrating a significant knowledge gap in the consent process. Prior direct exposure to autopsy during training was limited, with a substantial proportion (95%) of residents reporting that they had never attended an autopsy (Fig. 3).

Integration of the institutional workflow analysis with the consent audit and resident survey findings demonstrated that attrition in the hospital autopsy pathway occurred at multiple levels. Although consent initiation was infrequent, the most substantial loss occurred after consent had been obtained, where incomplete documentation, incorrect determination of indication, and minimal pathology involvement were associated with failure to convert consent into a performed autopsy. The resident survey further demonstrated that these process failures were accompanied by gaps in knowledge of autopsy indications and legal consent requirements, despite a strong perception of educational value.

In practical terms, several of the identified deficiencies represent correctable, real-time failures in the consent process. For example, incomplete documentation of required identifiers or missing clinician signatures frequently resulted in otherwise valid consents being unusable. Similarly, incorrect classification of cases as falling under medical examiner jurisdiction led to inappropriate exclusion from hospital autopsy pathways. In many instances, these issues could potentially be resolved through immediate review or clarification, such as direct communication between the clinical team and pathology personnel prior to finalizing the consent. These observations highlight that a subset of lost autopsies may be preventable through simple, point-of-care interventions within the workflow.

When baseline parameters were applied, the predictive model reproduced the observed institutional autopsy rate. Using 2025 data, a consent initiation rate of 6.8% and a consent-to-autopsy conversion rate of 8.3% yielded a modeled autopsy rate of 0.56%, which approximated the observed rate of 0.57%, validating the internal consistency of the model.

Scenario-based projections demonstrated that improvement in consent quality alone, modeled as an increase in conversion efficiency to 20% with unchanged initiation, would increase the autopsy rate to approximately 1.36%, representing a 2.4-fold increase over baseline. Improvement in clinician recognition of autopsy indications alone, modeled as an increase in consent initiation to 10% with unchanged conversion, would increase the autopsy rate to approximately 0.83%, corresponding to a 1.5-fold increase. A combined intervention targeting both initiation and conversion parameters was projected to increase the institutional autopsy rate to approximately 2.5%, representing a 4.4-fold increase compared with baseline (Table 3, Fig. 4).

**Table 3:** Scenario-based predictive model of hospital autopsy rate improvement. Projected autopsy rates derived from baseline initiation and conversion parameters after simulated improvements in consent quality and indication recognition. The combined intervention yields the greatest increase. The final row represents the 5% academic benchmark.

Scenario	What changes in the workflow	Consent initiation rate	Consent-to-autopsy conversion	Projected autopsy rate	Relative-fold increase vs. 2025
Baseline (observed 2025)	Current institutional performance	6.8%	8.3%	0.57%	1.0
Improved consent quality	Correct completion of forms and accurate indication determination	6.8%	20%	1.36%	2.4
Improved indication recognition (resident education)	More appropriate initiation of autopsy discussions	10%	8.3%	0.83%	1.5
Combined workflow intervention	Improved initiation and improved conversion	10%	25%	2.5%	4.4
Target academic benchmark	Sustained gains in both workflow steps	15%	33%	5.0%	8.8

**Fig. 4:**
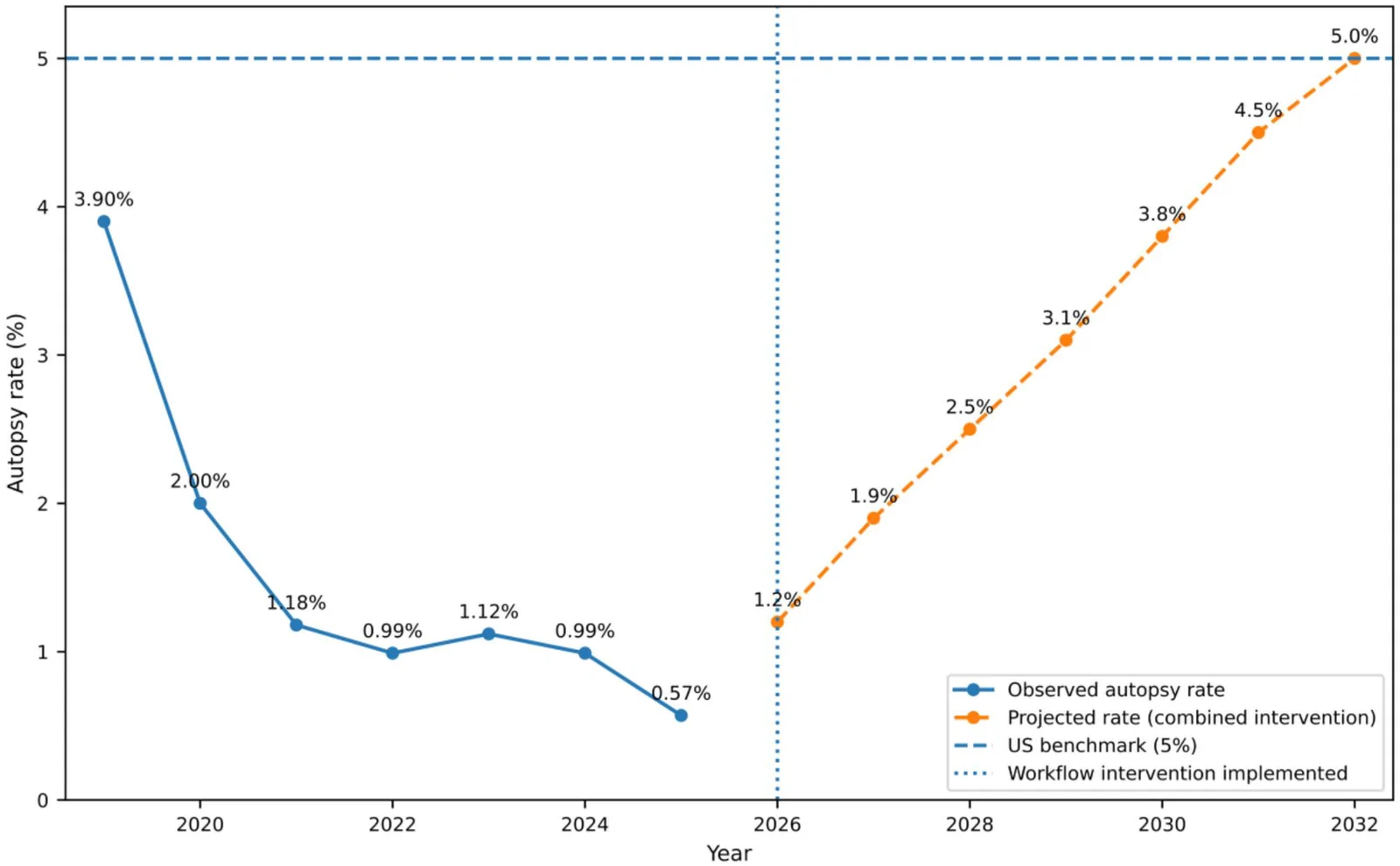
Observed and projected hospital autopsy rates. The blue line represents the observed institutional autopsy rate from 2019 to 2025. The orange line represents the projected autopsy rate following implementation of the combined workflow intervention beginning in 2026, based on improvements in consent initiation and consent-to-autopsy conversion rates derived from the predictive model. The horizontal dashed line indicates the 5% academic benchmark for hospital autopsy utilization. Values adjacent to each data point represent the corresponding autopsy rate for that year.

Scenario analysis further demonstrated that restoration of autopsy utilization to the contemporary academic benchmark of approximately 5% would require simultaneous increases in both consent initiation and consent-to-autopsy conversion, with multiple parameter combinations capable of achieving this target. These findings indicate that the observed decline is not attributable to a single point of failure but to modifiable deficiencies across sequential steps in the autopsy workflow.

## Discussion

The progressive decline in hospital autopsy utilization represents a global phenomenon affecting both community and academic medical centers, with contemporary autopsy rates in the United States consistently reported at approximately 5% of inpatient deaths and frequently lower in nonteaching institutions.^1^, ^2^, ^3^, ^4^ Against this background, the findings of the present study are especially notable. Despite being an academic teaching hospital, the institutional autopsy rate remained at or below 1% for most of the study period, falling to its lowest level of 0.57% in 2025. These values are substantially below those reported for comparable academic centers. When considered together with the persistently low consent initiation rate, our data indicate that the autopsy deficit begins early in the clinical workflow and is compounded by failures in subsequent steps.

By analyzing the autopsy as a multistep process consisting of death identification, consent initiation, and completion of the examination, this study provides a structural explanation for the observed decline.

Several academic institutions have proposed or implemented structured consent pathways that include standardized documentation, dedicated personnel trained in postmortem discussions, and early involvement of pathology services. In contrast, the consent process at our institution was largely dependent on clinical trainees without formal training or standardized workflow support. This difference likely contributes to the observed discrepancies in autopsy utilization. Unlike prior quality improvement efforts that assess autopsy rates as a single outcome, the approach used in the present study allows identification of specific points of failure within the workflow. Institution-specific factors, including the absence of a standardized consent workflow, limited pathology involvement, and lack of formal training in autopsy consent, likely contributed to the markedly lower autopsy rates observed compared with other academic centers.^8^^,^^9^

In this context, the choice of analytical framework becomes critical. A workflow-based analytical approach was selected because the autopsy process inherently consists of sequential, interdependent steps, each of which may contribute to overall failure. While root-cause analysis is valuable for identifying individual contributing factors, it does not readily quantify how failures at different stages affect overall performance. By contrast, this model enables estimation of the cumulative effect of deficiencies across multiple steps and supports scenario-based projections of targeted interventions.

Traditional explanations for the reduction in autopsy rates have focused on financial disincentives, the elimination of accreditation requirements, and the perception that advances in diagnostic imaging have rendered the autopsy obsolete^1^, ^2^, ^3^, ^4^, contemporary literature continues to emphasize the value of autopsy in modern medicine, particularly as a tool for diagnostic quality improvement and patient safety.^10^, ^11^, ^12^ While these factors contribute to the broader national trend, they do not fully account for the discrepancy between consent acquisition and autopsy completion observed in our institution. The marked decrease in the consent-to-autopsy conversion rate from 52% to 8% during the study period indicates that the greatest loss of cases occurred after consent had been obtained, representing an important and potentially modifiable point of failure. By comparison, our institution performed only one autopsy for every twelve consents obtained in 2025 (4:48), highlighting a substantial loss of potentially recoverable cases within the postconsent workflow.

The consent audit further clarified where cases were lost in the process. Approximately 40% of consent attempts were classified as low quality due to incomplete documentation, incorrect determination of indication, or internal inconsistencies in the form. This level of process failure contrasts with recommendations from academic pathology programs emphasizing standardized autopsy workflows, active pathology participation, and structured institutional support to optimize autopsy practice.^8^^,^^9^ These findings suggest that the autopsy should be considered a structured clinical procedure requiring trained personnel and a clear institutional process rather than an optional postmortem activity. The near absence of pathology involvement in the consent process (1.4% of cases) represents a critical and modifiable system deficiency. Institutions with structured autopsy programs have demonstrated that early and active involvement of pathology personnel in the consent process—including direct communication with clinical teams and, in some cases, with next of kin—can improve both consent quality and conversion to completed autopsy. These findings suggest that re-integrating pathology into the consent workflow represents a key, actionable opportunity for improving autopsy utilization.

The results of the resident survey provide a critical explanation for these process failures and identify the principal target for intervention. The vast majority of consents in our institution were completed by clinical residents or advanced practice providers, yet objective assessment demonstrated substantial variability in recognition of autopsy indications and in knowledge of the legal requirements for consent. This observation is consistent with prior studies showing that trainees are often responsible for initiating autopsy discussions despite receiving minimal formal education in this area.^7^^,^^10^ Importantly, although autopsy is widely viewed as an important educational and quality-assurance tool, it is rarely considered in clinical practice, suggesting that the main barriers are educational and structural rather than related to clinician perceptions.

In addition, recent literature continues to emphasize the importance of autopsy in modern clinical practice, particularly as a tool for diagnostic quality assurance and systems-based improvement.^11^^,^^12^

The frequent attribution of the decline in autopsy utilization to advances in imaging further illustrates the persistence of the misconception that modern diagnostic technologies have eliminated the need for postmortem examination. Multiple contemporary studies have demonstrated that major clinicopathologic discrepancies remain present in 10–30% of cases, even in the era of high-resolution imaging and advanced laboratory testing.^2^^,^^3^^,^^5^^,^^6^^,^^13^^,^^14^

In addition to its role in evaluating diagnostic accuracy, the autopsy continues to provide essential information for patient safety initiatives, identification of therapeutic complications, and detection of heritable conditions with important implications for surviving family members.^3^^,^^5^^,^^11^^,^^12^^,^^15^ Thus, the decline in autopsy utilization represents not only a loss of an educational tool but also a reduction in a critical component of institutional quality assurance.

A central strength of the present study is the integration of institutional mortality data, detailed consent-level analysis, and objective assessment of clinician knowledge into a single analytical framework. This approach enabled the development of a predictive model that connects specific steps in the autopsy process with measurable changes in autopsy utilization.

By expressing the autopsy rate as the product of consent initiation and consent-to-autopsy conversion, the model demonstrates that even modest improvements in individual workflow steps can produce substantial relative increases in autopsy performance. In the present study, improvement in consent quality alone was projected to more than double the institutional autopsy rate, whereas combined improvements in indication recognition and conversion efficiency were associated with a greater than fourfold increase. Importantly, the model also defines the parameter combinations required to achieve the contemporary academic benchmark of 5%, thereby providing a realistic and institution-specific roadmap for autopsy revitalization.

Beyond its descriptive value, the predictive model has direct operational implications. First, it permits objective estimation of the expected effect size of targeted interventions, allowing institutional leadership to evaluate the potential return on educational and process-based initiatives. Second, it provides a quantitative framework for prospective monitoring, in which annual measurements of consent initiation and conversion rates can be directly compared with projected values. In this way, the model allows autopsy utilization to be monitored as a continuous quality indicator rather than as a single institutional statistic. Third, the model shows that increasing autopsy utilization does not require immediate achievement of national benchmarks. Instead, incremental improvements in individual steps of the process can produce meaningful early gains.

The planned educational intervention directed at clinical resident physicians represents a logical and evidence-based response to these findings. Because trainees are responsible for the majority of consent discussions and documentation, they constitute the most effective target for intervention. Structured teaching focused on recognition of autopsy indications, legal consent requirements, and communication strategies with next of kin has been shown to increase both consent rates and autopsy completion in other institutions.^6^^,^^10^ In the present study, this intervention is expected to improve both initiation and conversion steps within the autopsy workflow. The predictive model provides a quantitative estimate of the magnitude of this effect; however, prospective longitudinal data will be required to determine whether the observed postintervention values follow the projected trajectory and whether these improvements can be sustained over time.

This study also highlights the importance of viewing the autopsy within the broader framework of graduate medical education. Direct exposure to autopsy during training was limited among survey respondents, a finding that has been consistently associated with reduced likelihood of requesting autopsies in clinical practice.^5^^,^^9^ Restoration of autopsy as an integral component of residency education has the potential not only to improve institutional autopsy rates but also to enhance diagnostic reasoning, clinicopathologic correlation, and systems-based practice.

Several limitations should be acknowledged. The study was conducted at a single academic center, and institutional practices may not be generalizable to all settings. The predictive model is based on deterministic scenario analysis and requires prospective validation. In addition, although the consent audit identifies documentation deficiencies, it does not fully capture competing clinical demands that may influence clinician behavior. Nevertheless, the comprehensive integration of workflow analysis, consent quality assessment, and trainee knowledge evaluation provides a reproducible framework for other institutions facing similar declines.

Future efforts will focus on the prospective implementation and evaluation of targeted interventions based on the findings of this study. This project has been designed as an ongoing quality improvement initiative, with data to be collected in the coming years to assess whether these interventions result in measurable improvements in autopsy utilization. Planned interventions include structured educational sessions for clinical residents, emphasizing recognition of appropriate autopsy indications, guidance on how to approach next of kin to effectively communicate the purpose, benefits, and expectations of autopsy, and strategies to improve documentation accuracy. In addition, efforts will be directed toward increasing the involvement of pathology personnel in the consent process to provide real-time support and ensure appropriate case selection. Longitudinal monitoring of consent initiation and consent-to-autopsy conversion rates will allow validation of the predictive model and assessment of the sustainability of these interventions over time.

These findings demonstrate that restoration of hospital autopsy practice is achievable through targeted, systems-based interventions focused on clinician education, structured consent workflows, and active pathology involvement. Prospective longitudinal follow-up will be essential to determine whether these measures translate into sustained increases in autopsy utilization and to validate the predictive model as an institutional quality improvement tool.

## Conclusion

Hospital autopsy underutilization at our institution is primarily driven by modifiable failures throughout the hospital autopsy workflow rather than by limitations in consent acquisition alone. A substantial proportion of potentially recoverable cases are lost after consent has been obtained. Targeted interventions focused on clinician education, standardized documentation, and increased pathology involvement provide a practical framework to improve autopsy utilization. This workflow-based model offers a reproducible strategy for restoring autopsy practice as a measurable component of diagnostic quality and patient safety.

## Declaration of competing interest

The authors declare that they have no known competing financial interests or personal relationships that could have appeared to influence the work reported in this paper.
