# Supplementary material for: Closing the gap: A workflow-based analysis of postconsent failure to restore hospital autopsy utilization
Source: Acad Pathol. 2026 Sep 23;13(4):100300. doi: 10.1016/j.acpath.2026.100300 (PMC13634380; doi:10.1016/j.acpath.2026.100300)
Supplement: Supplementary file 1 [file mmc1.docx]

**Supplemental Material 1.** Clinical resident physicians questionnaire and scenario-based assessment of autopsy indications.

**Anonymous Questionnaire**

**Introductory Statement:**
Participation in this survey is **voluntary**. You may choose not to answer any question or stop at any time without penalty. Your responses will be kept **anonymous**, and no personal identifiers will be collected. All data will be stored securely on password-protected systems accessible only to the research team. Results will only be reported in summary form to ensure your privacy and confidentiality. If you have any questions contact Alejandro Partida, MD ([alejapar@ttep.edu](mailto:alejapar@ttep.edu)).

**Please indicate your specialty:** ___________________________
**Please select your current level of training:**
☐ PGY-1	☐ PGY-2	☐ PGY-3	☐ PGY-4

1. Do you believe autopsies contribute to medical education and improving clinical practice? Why or why not?
A) Yes, they provide critical insights into disease mechanisms.
B) No, modern imaging has made them obsolete.
C) Only for rare diseases.
D) They have minimal educational value.

2. How often do you consider requesting an autopsy when a patient dies under your care?
A) Always.
B) Often.
C) Sometimes.
D) Rarely.

3. Do you think autopsy findings should be systematically reviewed in mortality and morbidity conferences? Why or why not?
A) Yes, it can improve patient care by identifying errors.
B) No, it would be too time-consuming.
C) Only for complex cases.
D) Only if there is a legal or financial interest.

4. Which of the following statements best describes the role of a clinician in discussing autopsy with a patient's family?
A) Only to provide basic information if the family asks about it.
B) To explain the medical, educational, and quality improvement benefits while being sensitive to cultural and emotional concerns, emphasizing that there is no financial burden to the family.
C) To discourage autopsies unless absolutely necessary for legal reasons.
D) To refer all autopsy discussions to the pathology department.

5. Which of the following factors is least likely to contribute to the decline in hospital autopsy rates in a county-based hospital?
A) Cultural and religious objections to autopsy.
B) Increased reliance on advanced diagnostic imaging.
C) Fear of revealing medical errors or discrepancies in clinical diagnosis.
D) High out-of-pocket costs for families.

6. What is the most accurate reason for the low autopsy rate (1%) at UMC El Paso?
A) High rates of accurately diagnosed terminal illnesses.
B) Cultural barriers, combined with a lack of physician referrals.
C) Financial barriers for patient’s relatives and cultural barriers.
D) Strict hospital regulations against autopsy.

7. Have you ever attended or observed an autopsy?
A) Yes, and it was a valuable experience.
B) Yes, but it did not impact my clinical practice.
C) No, but I would like to.
D) No, and I have no interest.

8. How should a clinician frame the discussion about autopsy with the family?
A) Emphasize the potential financial burden and legal implications.
B) Focus on the educational and public health benefits while acknowledging the emotional difficulty of the decision.
C) Avoid discussing autopsy unless the family explicitly asks about it.
D) Use technical medical terminology to convey the importance of the procedure.

9. Which of the following best describes the legal requirement for obtaining consent for a hospital-based autopsy?
A) No consent is required if the patient died within 24 hours of hospital admission.
B) Consent is always required from the legal next of kin, unless the death is under medical examiner jurisdiction.
C) Autopsy consent is not required if the patient had a signed advance directive allowing postmortem examination.
D) The treating physician can authorize the autopsy without family consent if the death was unexpected.

10. Which of the following best captures the potential benefits and drawbacks of performing an autopsy in a modern medical context?
A) Provides a definitive cause of death, identifies diagnostic errors, and contributes to medical education, but may reduce family trust in the healthcare team if discrepancies are found.
B) Primarily a tool for validating surgical outcomes and providing legal protection to hospitals.
C) Useful for confirming infectious disease diagnoses but rarely contributes to broader medical knowledge.
D) Valuable for teaching medical students basic anatomy but largely obsolete in modern practice.

11. Which of the following sets of criteria are most commonly used to determine the need for a hospital-based autopsy, excluding those automatically under medical examiner jurisdiction?
A) Deaths occurring within 24 hours of admission, deaths involving infectious disease, and deaths in high-risk surgical patients.
B) Unexpected or unexplained deaths, deaths with significant clinical uncertainty, and deaths potentially related to medical interventions.
C) Only deaths in patients under 40 years old or those with complex medical histories.
D) Any death in which the treating physician has unanswered clinical questions, regardless of the family's wishes.

12. Which of the following is the most comprehensive advantage of performing an autopsy in modern medical practice?
A) Provides a definitive legal record of death for risk management purposes.
B) Confirms clinical diagnoses, identifies diagnostic errors, reveals rare disease presentations, and contributes to quality improvement.
C) Primarily serves as a method for assessing the effectiveness of hospital infection control protocols.
D) Mainly used to identify potential organ donors for transplant programs.

13. A 54-year-old woman undergoes a routine laparoscopic cholecystectomy for symptomatic cholelithiasis. She is discharged the same day but is found unresponsive at home two days later. Despite resuscitation efforts, she is pronounced dead on arrival at the emergency department. Should an autopsy be performed in this case?

A) No, because the death is likely related to her known surgical history.
B) Yes, to identify potential postoperative complications such as pulmonary embolism, sepsis, or anesthesia-related complications.
C) No, because the likely cause of death is unrelated to the recent surgery.
D) No, unless the family requests it for legal purposes.

14. A 40-year-old man presents with rapidly progressive neurological decline over the course of three weeks, leading to coma and death. He had no significant past medical history and no known risk factors for infectious or neurodegenerative diseases. Should an autopsy be performed in this case?

A) No, because the cause of death is likely a stroke or other common neurological condition.
B) Yes, to identify rare neurological disorders, infections, or autoimmune conditions that might explain the rapid deterioration.
C) No, unless the family specifically requests it.
D) No, because the death occurred in a hospital with full neurological workup.

15. A 28-year-old male with no significant medical history is found dead in his apartment by a roommate. There are no signs of trauma, and his last known contact was the night before. Should an autopsy be performed in this case?

A) No, because the death was likely due to natural causes.
B) Yes, because sudden, unexplained deaths in young adults often involve potentially heritable or undiagnosed cardiac conditions.
C) No, unless the family requests it for legal reasons.
D) No, because toxicology is sufficient to determine the cause of death.

16. A 72-year-old male with metastatic pancreatic cancer and a Do-Not-Resuscitate (DNR) order passes away at home under hospice care. His death is consistent with his terminal diagnosis. Should an autopsy be performed in this case?

A) Yes, to confirm the exact cause of death as metastatic cancer.
B) No, because the death is clinically anticipated and consistent with the patient’s known terminal illness.
C) Yes, if the family requests it for legal reasons.
D) Yes, to identify any possible medical errors in his hospice care.

17. A 63-year-old woman with end-stage heart failure, severe chronic obstructive pulmonary disease (COPD), and metastatic breast cancer is transitioned to comfort care in the ICU after a long hospital stay. She dies peacefully within hours of extubation. Should an autopsy be performed in this case?

A) Yes, to confirm the cause of death as heart or lung failure.
B) Yes, if the family requests it for research purposes.
C) No, because the death is consistent with the patient’s known terminal condition and planned withdrawal of life support.
D) Yes, to rule out unexpected medical errors.

18. An 89-year-old woman with severe Alzheimer’s disease and multiple comorbidities, including recurrent aspiration pneumonia, dies in a long-term care facility. Her death is consistent with the natural progression of her disease. Should an autopsy be performed in this case?

A) Yes, to confirm the exact cause of death.
B) Yes, if the family requests it for legal reasons.
C) No, because the death is expected and consistent with the patient’s documented medical history.
D) Yes, to identify potential medical errors in her nursing home care.
