# Supplementary material for: Closing the gap: A workflow-based analysis of postconsent failure to restore hospital autopsy utilization
Source: Acad Pathol. 2026 Sep 23;13(4):100300. doi: 10.1016/j.acpath.2026.100300 (PMC13634380; doi:10.1016/j.acpath.2026.100300)
Supplement: Supplementary file 2 [file mmc2.docx]

# Supplemental Material 2.

# A. Hospital Autopsy Consent Form

***Section A***

Form must be completed for all Non-Medical Examiner Deaths.
If patient meets autopsy criteria, form will be waived and given to Legal Authorized Representative (LAR).

REQUIREMENTS:
- If the LAR requests the autopsy, all pertinent forms will apply.
- If the LAR requests a non-autopsy, physician to perform a service is required.
- LAR must fill out the Texas Department of State Health Services Permission Examination or Autopsy Consent.

1. Patient meets all or one of the criteria listed as follows:
 a. Criteria met – request for autopsy discussed with family	Yes / No
 b. Criteria not met – offer for autopsy discussed with family	Yes / No
 c. Patient does not meet criteria – family can request an autopsy

DECLINATION:
Physician please initial if request for autopsy was declined.
____ Family declined request / offer to perform an autopsy

Practitioner Signature: ______________________
Supervising Physician Name: __________________
Date/Time: _________________________________

CONSENT:
The attending physician or their designee will ensure the following written consent is obtained from the LAR when ordering an autopsy.

I request that the physicians and surgeons in attendance at University Medical Center of El Paso perform a complete autopsy on the remains of:
____________________ (decedent name)

I authorize the removal and retention of tissue fragments, specimens, or therapeutic purposes for such organs, tissues, and parts as needed for diagnosis.

Signature: ______________________	Date/Time: __________
Witness: ________________________ Date/Time: __________

PHYSICIAN AUTOPSY ORDER:
Physician Order: Please perform autopsy requested by:
☐ Family	☐ MD (consent)
Ordering Physician: __________________________
Medical Service: _____________________________

Physician Signature: _________________________
Supervising Physician Name: __________________
Fax Medical Record: __________________________

***Section B – Patient Identification Sticker***

# B. Institutional Medical Staff Criteria

Non-Medical Examiner Autopsy Consent and Order Form
Medical Staff Criteria

Check criteria that apply (need not meet all criteria to be eligible):

☐ Deaths in which autopsy may help to explain unknown or unexpected medical complications or laboratory findings
☐ All deaths in which cause of death is not known with certainty on clinical grounds
☐ Cases in which autopsy may help to clarify disease course or confirm diagnosis
☐ Unexpected deaths occurring during or following medical, dental, or surgical diagnostic procedures or therapies
☐ Deaths of patients who have participated in clinical trials
☐ Unexpected or unexplained deaths that are apparently natural and not subject to forensic medical jurisdiction
☐ Natural deaths that are subject to review by a forensic medical jurisdiction such as:
 - Deaths occurring in hospital within 24 hours of admission
 - Deaths in which the patient sustained or apparently sustained an injury
 - Deaths occurring in custody or under suspicious circumstances
☐ Deaths resulting from high-risk infectious and contagious diseases
☐ All neonatal and pediatric deaths
☐ Deaths of patients of any age when it is believed autopsy would disclose a known or suspected illness that also may have a bearing on survivors or recipients of transplant organs
☐ Deaths known or suspected to have resulted from environmental or occupational hazards
☐ Suspected congenital, structural, or chromosomal anomalies
☐ Suspected anatomic lesions
☐ Repeated second trimester losses
